# Secondary cardiac involvement in anti-SRP-antibody-positive myopathy: an 87-year-old woman with heart failure symptoms as the first clinical presentation

**DOI:** 10.1186/s12883-020-1599-5

**Published:** 2020-01-17

**Authors:** Arika Hara, Ryota Amano, Hiroaki Yokote, Masahide Ijima, Satoshi Zeniya, Toshiki Uchihara, Sawako Yada, Mayumi Masumura, Hidenobu Takei, Ichizo Nishino, Shuta Toru

**Affiliations:** 1 Department of Neurology, Nitobe Memorial Nakano General Hospital, 4-59-16 Chuo Nakano, Tokyo, 164-8607 Japan; 2 Department of Internal Medicine, Nitobe Memorial Nakano General Hospital, 4-59-16 Chuo Nakano, Tokyo, 164-8607 Japan; 3 Department of Neuromuscular Research, National Institute of Neuroscience, National Center of Neurology and Psychiatry, 4-1-1 Ogawahigashi-cho, Kodaira, Tokyo, 187-8502 Japan

**Keywords:** Necrotizing myopathy, Anti-signal recognition particle antibody, Colon carcinoma, Myocarditis, Non-sustained ventricular tachycardia

## Abstract

**Background:**

Necrotizing myopathy (NM) is defined by the dominant pathological feature of necrosis of muscle fibers without substantial lymphocytic inflammatory infiltration. Anti-signal recognition particle (SRP)-antibody-positive myopathy is related to NM. Anti-SRP-antibody-positive myopathy can comorbid with other disorders in some patients, however, comorbidity with malignant tumor and myopericarditis has still not been reported.

**Case presentation:**

An 87-year-old woman with dyspnea on exertion and leg edema was referred to our hospital because of suspected heart failure and elevated serum creatine kinase level. Upon hospitalization, she developed muscle weakness predominantly in the proximal muscles. Muscle biopsy and immunological blood test led to the diagnosis of anti-SRP-antibody-positive myopathy. A colon carcinoma was also found and surgically removed. The muscle weakness remained despite the tumor resection and treatment with methylprednisolone. Cardiac screening revealed arrhythmia and diastolic dysfunction with pericardial effusion, which recovered with intravenous immunoglobulin (IVIg) treatment.

**Conclusions:**

We reported the first case of anti-SRP-positive myopathy comorbid with colon carcinoma and myopericarditis. This case is rare in the point that heart failure symptoms were the first clinical presentation. The underlying mechanism is still not clear, however, physicians should be carefully aware of the neoplasm and cardiac involvement in anti-SRP-antibody positive-myopathy patients and should consider farther evaluation and management.

## Background

Necrotizing myopathy (NM) is defined by the dominant pathological feature of necrosis of muscle fibers without substantial lymphocytic inflammatory infiltration. Currently, anti-signal recognition particle (SRP) and anti-hydroxy-3-methylglutaryl-CoA reductase (HMGCR) autoantibodies are reported to have a close association with NM [1].

SRP is a cytoplasmic ribonucleoprotein complex of six polypeptides and a specific RNA sequence labeled 7S [2]. Anti-SRP antibody was first reported by Reeves et al. in 1986 in a single patient diagnosed with polymyositis [3]. After this observation, this antibody has been reported to be found in approximately 5–20% of inflammatory myopathy cases [4–7]. Patients with anti-SRP antibody were found to have an extremely high level of serum creatine kinase (CK) and severe muscle weakness. They often require aggressive and prolonged immunomodulation [5]. In patients with anti-SRP-antibody-positive myopathy, skin rash, interstitial lung disease, arthritis, and cardiac involvement are reported as extramuscular features [6, 8–11]. However, recent data suggest that the cardiac involvement rate is relatively low [5, 6, 12, 13]. Furthermore, large case series have reported that there is no association of anti-SRP-antibody-positive myopathy and malignancy [4–6, 12–15].

We report the case of a patient with anti-SRP-antibody-positive NM who presented with heart failure as the initial symptom, which did not improve by the resection of colon carcinoma and high-dose methylprednisolone but improved remarkably after intravenous immunoglobulin (IVIg) therapy.

## Case presentation

An 87-year-old woman with dyspnea on exertion and lower limb pitting edema was referred to our hospital because of suspected heart failure. She also noticed watery melena 3 months before and slight fever and bilateral lower limb pitting edema 2 weeks before admission. She had been diagnosed as having hypertension and had been taking calcium antagonist and angiotensin receptor blocker. She had no history of taking statin-based medicine.

On admission, her vitals were as follows: body temperature, 38.8 °C; heart rate, 70 beats/min; respiration rate, 25 breaths/min; and blood pressure, 186/91 mmHg. Her oxygen saturation was 97% at room air.

Physical examination revealed bilateral lower limb pitting edema and a Levine 2/6 systolic regurgitation murmur at the apex. She had no signs or symptoms that indicated dermatomyositis (i.e., muscle grasping pain, Gottron’s papule, and heliotrope rash). Neurological examinations revealed proximal limb motor weakness (manual muscle test score, 3–4/4), mainly in the neck flexor, deltoid, iliopsoas, gluteus maximus, and quadriceps muscle. We did not observe cranial nerve palsies, muscle pains, fasciculation, sensory disturbances, cerebellar ataxia, or abnormal deep tendon reflexes.

Laboratory examinations revealed high serum levels of the muscle-related enzymes (CK, 4195 mg/dL and CK-Mb, 191.8 ng/mL) and brain natriuretic peptide (285.9 pg/mL). She also showed anemia (hemoglobin level, 10.2 g/dL), hyponatremia (Na, 126 mEq/L), and thyroidal dysfunction (thyroid-stimulating hormone, 8.3 μIU/mL; free T3, 1.5 pg/mL; and free T4, 1.0 ng/dL). Renal function, glycometabolism, and other myocardial markers were within the normal ranges.

**Fig. 1:**
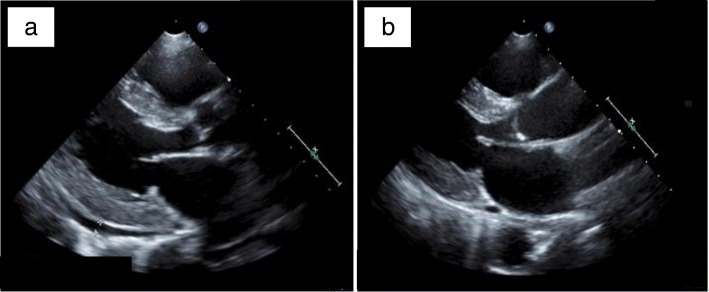
Pretreatment transthoracic echocardiogram showing diastolic left ventricular dysfunction (*E*/*e*′ = 19.2) with a well-preserved ejection fraction (73%), along with left ventricular wall hypertrophy (end-diastolic intraventricular septal thickness, 13 mm) and pericardial effusion (**a**). After the treatment, the diastolic left ventricular dysfunction (*E*/*e*′ = 11.7), left ventricular wall hypertrophy (IVSth = 11 mm), and pericardial effusion improved (**b**)


Electrocardiography revealed sinus rhythm with the narrow QRS complex. The p-wave morphology was biphasic. The findings met the criteria of left ventricular high voltage without ST-segment abnormalities. Transthoracic echocardiography revealed diastolic left ventricular dysfunction (*E*/*e*′ = 19.2) with well-preserved ejection fraction (73%), along with left ventricular wall hypertrophy (end-diastolic intraventricular septal thickness, 13 mm) and pericardial effusion (Fig. 1a; Additional file 1, 2 and 3).

**Additional file 1.** Echocardiography on admission: Parasternal long-axis view



**Additional file 2.** Echocardiography on admission: Apical four-chamber view



**Additional file 3.** Echocardiography on admission: Parasternal short-axis view


Chest computed tomography (CT) revealed bilateral pleural fluid retention and pericardial effusion without lung congestion indicating right heart failure. An abdominopelvic CT scan showed wall thickening of the Ra region on the rectum and lateral lymphadenopathy, indicating advanced rectal cancer. Colonoscopy revealed a type I progressive rectal tumor located 5 cm proximal to the anal verge and a type I primary rectal tumor located 18 cm proximal to the anal verge. In addition, primary colon cancer was found in the ascending colon. Histological results confirmed triple adenocarcinomas of the rectum and ascending colon with no evidence of metastasis.

Femoral magnetic resonance imaging (MRI) of the short T1 inversion recovery (STIR) sequence revealed high-intensity lesions in the left vastus lateralis muscle. A needle electromyographic study (nEMG) showed fibrillation potential, positive sharp wave, and poly-phasic motor unit potential on the right vastus lateralis muscle but not on the right tibialis anterior muscle.

**Fig. 2:**
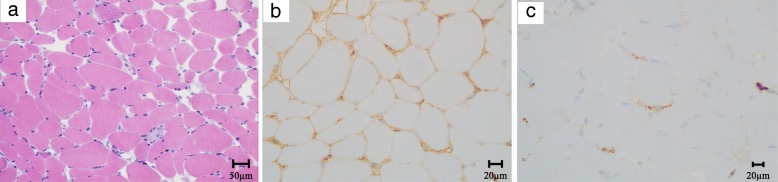
Histopathological findings of the left vastus lateralis muscle. Hematoxylin and eosin staining showed muscle fibers of various sizes accompanied by necrotic and regenerating fibers, and inflammatory cell infiltration (**a**). Almost all the fibers were immunopositive for major histocompatibility complex (MHC) class I (**b**). Membrane attack complex (MAC) equivocally deposits in some muscular surfaces (**c**)

A muscle biopsy from her left vastus lateralis muscle revealed muscle fibers of various sizes accompanied by necrotic and regenerating fibers along with immunopositivity for major histocompatibility complex class I, whereas only slight to mild inflammatory cell infiltration was noted around the muscle fibers (Fig. 2a, b). Furthermore, membrane attack complex (MAC) equivocally deposited on some muscular surfaces (Fig. 2c). The patient’s serum was positive for anti-SRP antibodies, but negative for anti-HMGCR antibodies and other myositis-specific antibodies. On the basis of the pathological findings and additional serum examinations, she was diagnosed as having anti-SRP antibody-positive NM.

The muscle weakness progressed rapidly after admission. The patient underwent laparoscopic lower anterior/ileocecal resection of the rectal/colon cancer and received high-dose methylprednisolone (1000 mg/day × 3 days intravenously) as initial treatment. Prednisolone (50 mg/day [1 mg/kg] orally) was administered after the initial treatment. Although her serum CK level decreased with the prednisolone therapy, her muscle weakness did not improve. After initiating IVIg therapy (400 mg/[kg·day]), her muscle weakness recovered remarkably. She was discharged 94 days after admission. Her prednisolone dose was gradually tapered to 8 mg/day without relapse 12 months after discharge.

For heart failure, administration of diuretics was initiated immediately after admission. During the in-hospital treatment, paroxysmal supraventricular tachycardia, atrial fibrillation, and non-sustained ventricular tachycardia were observed. Parallel with the improvement in muscle strength, her arrhythmia, diastolic left ventricular dysfunction, and pericardial effusion also improved (Fig. 1b; Table 1; Additional files 4, 5 and 6).

**Table 1:** Echocardiography pre and post treatment

	Pre-treatment	Post-treatment
IVSth (mm)	13	11
PWth (mm)	11	9
LVDd (mm)	46	42
LVDs (mm)	26	22
EF (%)	73	79
FS (%)	43	48
E/e’	19.2	11.7
Pericardial effusion	+	–

*IVSth* Thickness of interventricular septum, *PWth* Thickness of left ventricular posterior wall, *LVDd* Left ventricular end-diastolic diameter, *LVDs* Left ventricular end-systolic diameter, *EF* Ejection fraction, *FS* Fractional shortening


**Additional file 4.** Echocardiography after IVIg tharapy: Parasternal long-axis view



**Additional file 5.** Echocardiography after IVIg therapy: Apical four-chamber view



**Additional file 6.** Echocardiography after IVIg therapy: Parasternal short-axis view


**Fig. 3:**
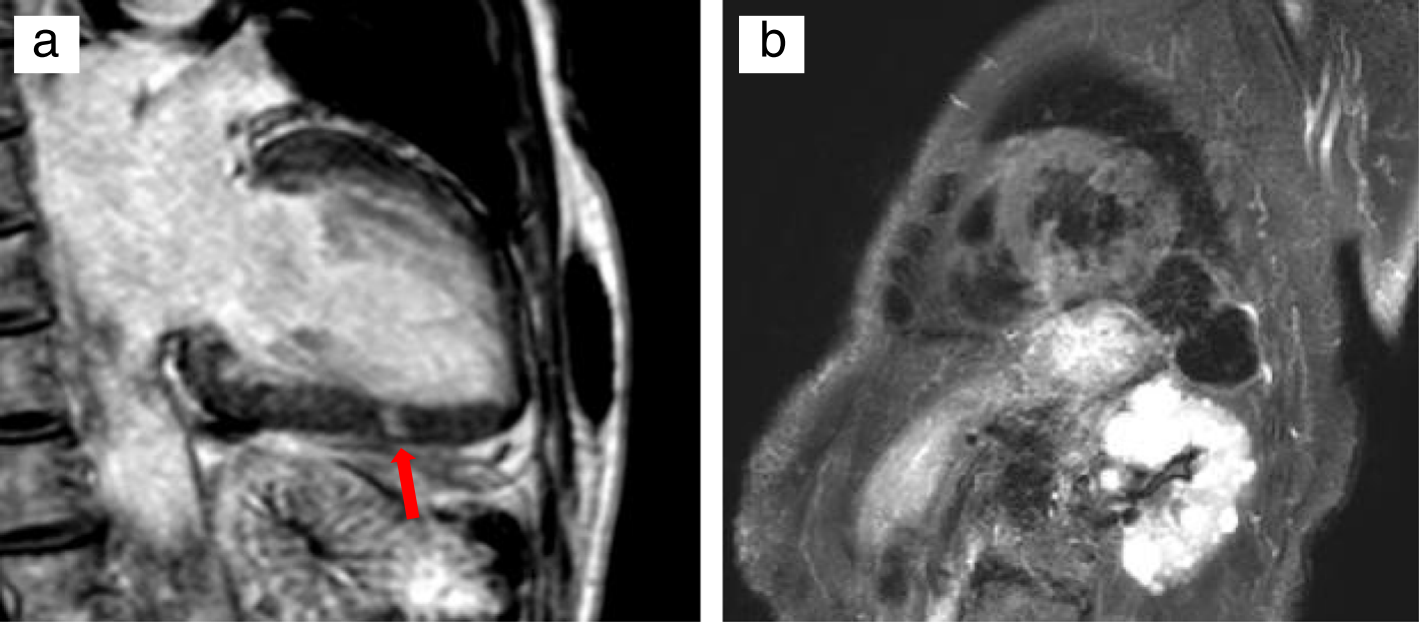
Contrast-enhanced cardiac magnetic resonance image taken 5 months after hospital discharge showing spotty late gadolinium enhancement in the middle inferior wall (**a**) and no high-intensity area in the T2-weighted image (**b**)

The contrast-enhanced cardiac MRI performed 5 months after discharge revealed a spotty late gadolinium enhancement in the middle inferior wall of the left ventricle. T2-weighted imaging did not show any high-intensity area, suggesting a post-myocarditis change (Fig. 3).

## Discussion and conclusions

Anti-SRP-antibody-positive myopathy, which usually leads to severe proximal muscle weakness, is also known to involve extramuscular symptoms such as dysphagia, respiratory disorders, and cardiac disorders [1, 14].

Comparative studies have shown that there is no significant difference in prevalence of malignancies between anti-SRP-antibody-positive myopathy patients and others [4–6, 12–15]. Allenbach et al. reported in analysis of 49 patients with anti-SRP-antibody positive myopathy that there is no increased incidence of malignancy [16]. However, only a few cases of anti-SRP-antibody-positive myopathy associated with malignancy have been reported so far [17]. We considered that our case shows the putative association of malignancies and anti-SRP-antibody-positive myopathies.

The frequency of cardiac involvement was high in past reports published around 1990. Targoff et al. reported on 4 of 13 anti-SRP-antibody-positive myopathy patients with cardiac involvement such as arrhythmia, heart failure, and cardiac fibrosis [4]. Moreover, Love et al. reported that all their seven cases showed palpitations [15]. Conversely, another report published after 2000 showed a relatively low or almost the same incidence rate as in the general population. Hengstman et al. reported in 2006 that < 20% of patients with anti-SRP-antibody-positive myopathy showed heart failure [12]. Furthermore, Suzuki et al. reported cardiac involvement in only 2 of 100 patients with anti-SRP-antibody-positive myopathy [13]. Currently, the prevalence of cardiac involvement in patients with anti-SRP-antibody-positive myopathy is still controversial.

We identified six case series reporting on patients with myopericarditis with anti-SRP-antibodies [9–11, 18–20]. Three of the cases showed pericardial effusion like the present case [10, 11, 18], and two showed diastolic left ventricular dysfunction [18, 20]. Three reports showed that the initial symptoms were related to heart failure [10, 11, 19]. Furthermore, Takeguchi-Kikuchi et al. recently demonstrated that anti-SRP antibody-positive NM is associated with cardiomyopathy, which was demonstrated by myocardial biopsy, cardiac MRI, and fluorodeoxyglucose-positron emission tomography [18]. In that case, the electrocardiogram demonstrated left ventricular hypokinesis, pericardial effusion, and diastolic dysfunction, similar to that in our case. These three findings can be related to myocarditis with anti-SRP-antibody-positive myopathy, as reported previously [10, 11, 18, 20]. On the basis of these reports, the prevalence of cardiac involvement in anti-SRP-antibody-positive myopathy can be higher than expected.

In the present case, the malignancy was T3N1M0 by TNM classification, and the excision margin of the pathological specimen removed by the surgery was negative. Therefore, the possibility of myocardial metastasis and pericardial metastasis was extremely low. After IVIg therapy, the diastolic left ventricular (LV) function, LV wall thickness, and pericardial effusion improved. In addition, her arrhythmia also disappeared. If contrast-enhanced cardiac MRI is available at the time of the initial treatment, the myocarditis can be more precisely diagnosed. Although there might be a possibility of the effect after the diuretics and cardioprotective treatment, the findings of cardiac MRI and the fact of the improvement of cardiac findings suggest the association between the myocarditis and the anti-SRP-antibody-positive myopathy. Contrast-enhanced cardiac MRI after the acute phase is still meaningful.

In conclusion, we report the case of a patient with NM complicated by myocarditis. The initial presentation of NM can be cardiac symptoms. On the contrary, the cause of heart failure may be masked by the muscle weakness. For patients with NM, cardiac screening, evaluation, management, and follow-up should be considered. Early diagnosis and treatment of both myopathy and myocarditis can definitely improve the clinical outcomes of anti-SRP-antibody-positive myopathy.

## Data Availability

All data related to this case report are contained within the manuscript.

## Abbreviations

CK: Creatine kinase
CT: Computed tomography
HMGCR: Hydroxy-3-methylglutaryl-CoA reductase
IVIg: Intravenous immunoglobulin
LV: Left ventricular
MAC: Membrane attack complex
MRI: Magnetic resonance imaging
nEMG: Needle electromyographic
NM: Necrotizing myopathy
SRP: Signal recognition particle
STIR: Short T1 inversion recovery
